# Fetal brain 11β-hydroxysteroid dehydrogenase type 2 selectively determines programming of adult depressive-like behaviors and cognitive function, but not anxiety behaviors in male mice

**DOI:** 10.1016/j.psyneuen.2015.05.003

**Published:** 2015-09

**Authors:** Caitlin Wyrwoll, Marianne Keith, June Noble, Paula L. Stevenson, Vincent Bombail, Sandra Crombie, Louise C. Evans, Matthew A. Bailey, Emma Wood, Jonathan R. Seckl, Megan C. Holmes

**Affiliations:** a UoE/BHF Centre for Cardiovascular Science, University of Edinburgh, EH16 4TJ, United Kingdom; b Centre for Cognitive and Neural Systems, University of Edinburgh, EH8 9JZ, United Kingdom; c School of Anatomy, Physiology & Human Biology, The University of Western Australia, 35 Stirling Highway, Crawley, WA 6009, Australia

**Keywords:** Glucocorticoids, Developmental programming, Affective behaviors, Brain 11β-HSD2

## Abstract

Stress or elevated glucocorticoids during sensitive windows of fetal development increase the risk of neuropsychiatric disorders in adult rodents and humans, a phenomenon known as glucocorticoid programming. 11β-Hydroxysteroid dehydrogenase type 2 (11β-HSD2), which catalyses rapid inactivation of glucocorticoids in the placenta, controls access of maternal glucocorticoids to the fetal compartment, placing it in a key position to modulate glucocorticoid programming of behavior. However, the importance of the high expression of 11β-HSD2 within the midgestational fetal brain is unknown. To examine this, a brain-specific knockout of 11β-HSD2 (HSD2BKO) was generated and compared to wild-type littermates. HSD2BKO have markedly diminished fetal brain 11β-HSD2, but intact fetal body and placental 11β-HSD2 and normal fetal and placental growth. Despite normal fetal plasma corticosterone, HSD2BKO exhibit elevated fetal brain corticosterone levels at midgestation. As adults, HSD2BKO show depressive-like behavior and have cognitive impairments. However, unlike complete feto-placental deficiency, HSD2BKO show no anxiety-like behavioral deficits. The clear mechanistic separation of the programmed components of depression and cognition from anxiety implies distinct mechanisms of pathogenesis, affording potential opportunities for stratified interventions.

## Highlights

• Aberrant exposures to glucocorticoids during fetal life programme the risk of psychiatric disease in offspring.
• Placental 11β-HSD2 protects the fetus from maternal glucocorticoids.
• This study investigates the role of 11β-HSD2 in the fetal brain.
• Deletion of 11β-HSD2 in the fetal brain causes depressive-like and cognitive dysfunction in adults.
• Thus fetal brain 11β-HSD2 protects against programming of adult brain function.

## 1 Introduction

Lower birth weight, a marker of an adverse fetal environment, associates with neuropsychiatric abnormalities in later life (Khashan et al., 2011). Similarly, maternal exposure to stress during pregnancy associates with an increased offspring prevalence of psychiatric disease and cognitive dysfunction (Weinstock, 2008). Both may be underpinned by elevated maternal glucocorticoids reaching the fetus at inappropriate times during development, retarding fetal growth and altering the timing of cessation of cellular (neuronal and glial) proliferation and terminal differentiation (Cottrell and Seckl, 2009). Maternal glucocorticoid administration causes behavioral and cognitive abnormalities in the offspring in a range of species (Pryce et al., 2011; Welberg et al., 2000), including humans (Pesonen et al., 2009; Trautman et al., 1995; Yeh et al., 2004).

To maintain the fetus in a low glucocorticoid environment in the face of 10-fold higher maternal blood concentrations, the placenta expresses the potent glucocorticoid-metabolising enzyme, 11β-hydroxysteroid dehydrogenase type 2 (11β-HSD2) which catalyses the rapid conversion of active cortisol and corticosterone to inert 11-keto forms (cortisone, 11-dehydrocorticosterone) (Brown et al., 1996a; Burton et al., 1996). This affords a relative enzymic ‘barrier’ which effectively excludes ∼80% of maternal active glucocorticoids from the fetal compartment (Benediktsson et al., 1997). Placental 11β-HSD2 is reduced with maternal malnutrition, stress or illness in rodent models and humans (Cottrell et al., 2014; Mairesse et al., 2007; O’Donnell et al., 2012); it is bypassed by poor substrates such as dexamethasone and betamethasone.

Mice globally deficient in 11β-HSD2 (11β-HSD2^−/−^) have low birth-weight and exhibit increased anxiety and depressive-like adult behaviors (Holmes et al., 2006a; Wyrwoll and Holmes, 2012). Similarly, the offspring of pregnant rats administered carbenoxolone, a soluble derivative of the potent 11β-HSD inhibitor in liquorice, show anxiety-related behaviors as adults (Welberg et al., 2000). In pregnant women, greater ingestion of liquorice in foodstuffs associates with reduced cognition and increased anxiety and attentional deficits in offspring (Raikkonen et al., 2009, 2010). Such data are consistent with the hypothesis that placental 11β-HSD2 protects the developing fetus from maternal glucocorticoids and their effects to programme the fetal brain. Furthermore, 11β-HSD2^−/−^ mice also have smaller, dysfunctional placentas (Wyrwoll et al., 2009) and hence glucocorticoid effects directly on the placenta rather than just the fetus may underpin, in part, the programmed phenotype. Indeed both fetal growth restriction and placental insufficiency are consistent findings across models of glucocorticoid programming.

However, there is also substantial fetal expression of 11β-HSD2, notably in the human, rat, mouse and pig fetal brain (Brown et al., 1996a, 1996b). This shows locus-specific patterns of late developmental inactivation that appear to correlate with the entry of brain regions into terminal differentiation pathways that are induced by glucocorticoids (Brown et al., 1996a; Holmes et al., 2006b). Moreover, at least in rodents, though fetal programming by maternal malnutrition is associated with elevated fetal glucocorticoid levels, these appear of fetal adrenal rather than maternal circulation origins (Cottrell et al., 2012). This might suggest a role for fetal tissue catabolism of glucocorticoids in modulating brain development. To interrogate this we generated a brain-specific knockout of 11β-HSD2, effective from early fetal life, and determined the effects on neurodevelopment, affective and cognitive behaviors, neurotransmitter levels and central gene expression.

## 2 Materials and methods

### 2.1 Mice

Brain-specific knock down of 11β-HSD2 (HSD2BKO) mice were generated by crossing 11β-HSD2^flx/flx^ mice (Artemis Pharmaceuticals, Cologne, Germany) with a Cre mouse line driven by the Nestin promotor (Tronche et al., 1999), which is expressed in proliferating CNS cells. For more information on the breeding schedule, housing conditions and verification of control mice *see Supplementary Information (SI) Methods.* Studies were carried out in strict accordance with the UK Home Office Animals (Scientific Procedures) Act, 1986 and the European Communities Council Directive of 24 November 1986 (86/609/EEC) and were assessed by the University of Edinburgh Ethical Review Committee. All experiments were performed blind to genotype on male mice using littermate controls (CON). Female offspring were not assessed in this study due to the potential confounding effects of the oestrous cycle.

### 2.2 Tissue collections

Prenatal tissue collections were taken at E12.5, E15.5 or E17.5 to assess 11β-HSD2 activity (Brown et al., 1993), corticosterone levels, and/or gene expression in CON and HSD2BKO fetal and placental tissues (see *SI Methods*). At postnatal day (P) 21, cerebellum were dissected and assessed for morphological changes as described by Holmes et al. (2006b). Adult brains and adrenals were collected and processed as described in *SI Methods*.

### 2.3 Neurodevelopment markers

To determine the rate of development of postnatal functional neural maturation, negative geotaxis and timing of eye opening were assessed at P7 and P14 respectively (Holmes et al., 2006b). For negative geotaxis, pups were removed from their mothers and placed on an inclined plane of approximately 30° with their head facing downwards. The number of mice which completed a reflex turn to face up the slope within a 1 min period was recorded.

### 2.4 Behavioral testing

All behavioral tests were performed during the light period between 08:00 and 13:00 h on male mice between 3 and 4 months of age. When appropriate, all behavioral tests were recorded using the Limelight™ video tracking system (Actimetrics Inc., IL, USA) to enable analysis.

*Elevated plus maze*: Exploratory behavior of the elevated plus maze was assessed for 5 min (Holmes et al., 2006a) with decreased exploration of the open arms/total movement indicative of anxiety-like behavior. *Open field:* Exploration of the open field was assessed as previously described (Holmes et al., 2006a), and mice were allowed to explore for 5 min. Decreased exploration of the center was indicative of anxiety-like behavior. *Tail suspension:* The tail suspension test was adapted from (Steru et al., 1985). Mice were individually suspended from a shelf above a table using an adhesive tape placed 0.5 cm from the tip of the tail. Mice were scored every 5 s for 6 min as either struggling or immobile, with immobility an indicator of depressive-like characteristics. *Forced swim test:* Mice were placed for 6 min in a 5 L glass beaker filled to a depth of 15 cm (3 L) with tepid (22–25 °C) tap water. At 5 s intervals, mice were scored as either swimming/struggling or climbing versus floating, with floating an indicator of depressive-like behavior. *Novelty-induced hypophagia (NIH):* To conduct the NIH study methods were adapted from Mombereau et al. (2010). Briefly, singly housed mice were initially trained to consume chocolate chips from a plastic petri dish in their home cage for 5 days and on the 6th day, the test paradigm was carried out in a novel environment for 5 min and distance travelled and latency to eat were recorded, with a longer latency to eat an indicator of depressive-like behavior. *Morris water maze test:* The Morris water maze was used to assess behavior associated with learning and memory of the CON and HSD2BKO mice. Procedures were followed as detailed by Yau et al. (2001). Latency to escape the water is a measure of learning ability. At the end of day five, mice were given a probe trial whereby the platform was removed and the time and direction of swim search was considered to be an indicator of memory retention. *Object Recognition*: The procedure for these tasks was informed by previous publications (Cost et al., 2014). Mice were acclimatised over 5 days to the behavioral arena and two objects. Object recognition consisted of a sample and a test phase, whereby in the sample phase, each animal was allowed to explore 2 identical novel objects for 5 min, followed by a 1 h interval before the test phase. Here one object was replaced by a completely novel object. The mouse was then allowed to explore the objects/box for 5 min and the time spent actively exploring each object was recorded. *Object in Place:* Each mouse was acclimatised to the test box containing 4 objects over a 5 day period. The sample phase of the experiment involved the mouse being placed in the box equipped with four different novel objects for 7 min. Time spent exploring each of the 4 objects was recorded. Following a delay of 5 m or 1 h the mice were returned to the box for the test phase of 3 min, where 2 of the objects had exchanged positions. The amount of time spent exploring each object was recorded. The Discrimination ratio was calculated as an index of memory performance (time at novel − time at familiar)/(time at novel + time at familiar). For each mouse, the mean discrimination ratio for each task was obtained by calculating the mean of the discrimination ratios for the 2 trials on that task.

### 2.5 Rotarod

To assess general agility and balance of the mice, they were individually placed onto a rotating rod, which gradually increases in rotation velocity. The length of time the mice stayed on the rod once rotation commenced was recorded.

### 2.6 Food and water intake

CON and HSD2BKO mice were individually housed in food and drink monitoring cages (TSE Systems, Bad Homburg, Germany) and their food and drink intake measured every minute using Drink (TSE Systems, Bad Homburg, Germany). Mice were allowed to acclimatise for three days and average hourly intakes were calculated for four 24 h cycles.

### 2.7 Assessment of HPA axis function

Blood samples were collected from singly housed adult animals by tail nick at 0700 h and 2000 h in order to assess nadir and peak circulating corticosterone levels. In a separate cohort of animals, HPA axis response to acute stress was assessed by placing animals into a restraint tube for 40 min. Blood samples were collected by tail nick before animals were placed into a restraint tube (T0), 30 min into the restraint tube (T30) and one hour post-restraint stress (T90). Blood was collected into EDTA-coated Microvette tubes (Sarstedt, Numbrecht, Germany) and centrifuged, and plasma stored at −20 °C until corticosterone determination by radioimmunoassay as described previously (Holmes et al., 1997).

### 2.8 CNS expression analyses

In situ hybridisation was performed as described previously (Harris et al., 2001). Mouse brain sections were assessed for serotonin receptor 1A (*Htr1a; hippocampus, cortex, baserolateral amygdala, dorsal and medial Raphe nuclei*), tryptophan hydroxylase 2 (*Tph2; dorsal and medial Raphe nuclei*), serotonin transporter (*Slc6a4; dorsal and medial Raphe nuclei*), dopamine receptor D2 (*Drd2; caudate putamen, nucleus accumbens core and shell*), dopamine transporter (*Slc6a3; ventral tegmental area, substantia nigra*), mineralocorticoid receptor (*Nr3c2; hippocampus*), glucocorticoid receptor (*Nr3c1; paraventricular nucleus (PVN), hippocampus*) and corticotrophin-releasing hormone (*Crh; PVN*). Expression of *Hsd11b1* and *Hsd11b2* in fetal brain, and *Nr3c1* and *Nr3c2 in adult hippocampi* were assessed by quantitative real-time RT-PCR (Wyrwoll et al., 2009). For more detail see *SI Methods.*

### 2.9 High performance liquid chromatography (HPLC)

Brains were dissected into cortex, diencephalon, hippocampus and hindbrain. The dissected samples were weighed and homogenised in 200 μl 0.1 M perchloric acid (PCA), sonicated twice for 10 s and spun (10,000 × *g*, 5 min, 4 °C). The supernatant was processed for HPLC measurement of monoamines and their metabolites as described in *SI Methods*.

### 2.10 Cardiovascular parameters

Mean arterial blood pressure, urinary sodium excretion, sodium potassium ratio and haematocrit were measured in anaesthetised CON and HSDBKO mice according to Hunter et al. (2014).

### 2.11 Statistical analysis

All data are expressed as group means ± SEM. Statistical analyses were conducted with Graphpad Prism v5 or 6.0d, using either unpaired *t*-test, one- or two-way ANOVA and where appropriate, this was followed by a post hoc Tukey's HSD test. Corticosterone levels obtained from the restraint stress studies were analysed using two-way ANOVA with repeated measures. Values are considered statistically significant at *p* < 0.05.

## 3 Results

### 3.1 Verification of 11β-HSD2 removal from the developing brain

**Figure 1:**
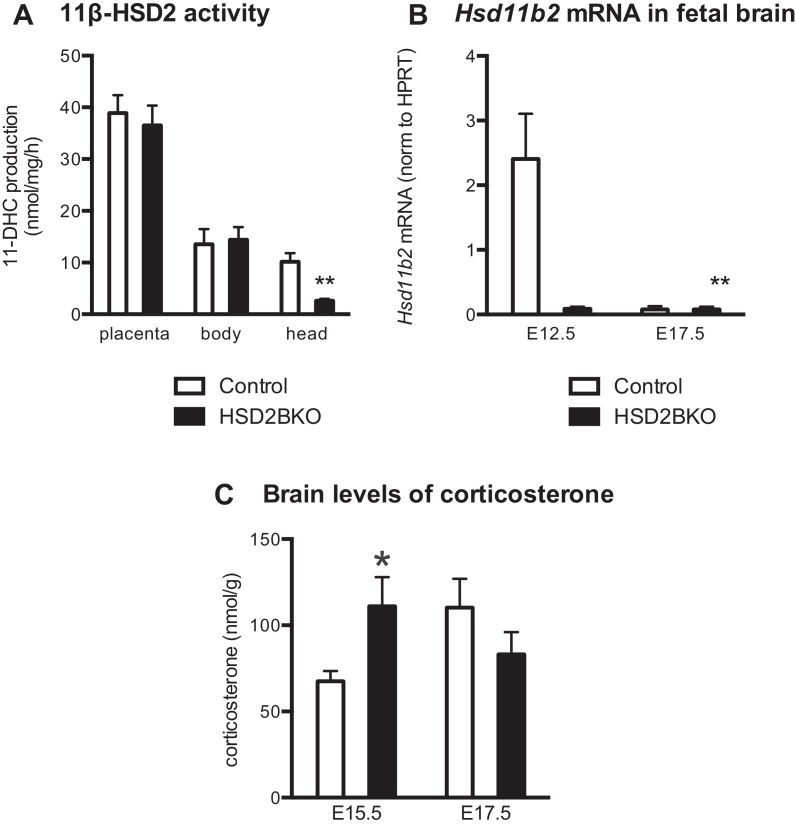
(A) Feto-placental 11β-HSD2 activity at E12.5. 11β-HSD2 activity is reduced in the fetal head of E12.5 HSD2BKO mice (*n* = 6) in comparison to controls (*n* = 6). Within the placenta and the remainder of the fetal body 11β-HSD2 activity was unaltered by phenotype (*n* = 5–7). **p < 0.005 compared to controls. (B) *Hsd11b2* mRNA levels in fetal brain. *Hsd11b2* mRNA levels are reduced dramatically at E12.5 in HSD2BKO (*n* = 5) compared to controls (*n* = 5) while minimal expression is seen in either genotype at E17.5 (*n* = 4–5). 2-Way ANOVA reveals significant effects of genotype (*F*(1,15) = 9.697, *p* = 0.007), age (*F*(1,15) = 9.519, *p* = 0.0075) and interaction (*F*(1,15) = 9.498, *p* = 0.0076). (C) Brain corticosterone content at E15.5 and E17.5. Corticosterone content is increased in brain of E15.5 HSD2BKO (*n* = 10) compared to controls (*n* = 9). *p < 0.05 compared to Controls.

Brain-specific knock down of 11β-HSD2 substantially reduced (75%) 11β-HSD2 activity (Fig. 1A) and (96%) *Hsd11b2* mRNA (Fig. 1B) in the fetal head at E12.5 (the peak of gestational 11β-HSD2 expression) in comparison to wild-type control (CON) littermate fetuses. By E17.5, near the end of gestation, 11β-HSD2 activity was dramatically decreased in both genotypes (CON: 0.5222 ± 0.0943 nmol/mg/h, *n* = 6; HSD2BKO: 0.2466 ± 0.0699 nmol/mg/h, *n* = 5) however a decreased activity was still observed in the HSD2BKO brains (*p* = 0.05). *Hsd11b2* mRNA levels were also extremely low at E17.5 with no difference between genotype at this stage (Fig. 1B). However, HSD2BKO had no effect on 11β-HSD2 activity in fetal body or placenta (Fig. 1A). 11β-HSD2 activity was not altered in HSD2BKO adult kidneys (CON 64.36 ± 10.61 nmol 11-dehydrocorticosterone (11DHC)/mg/h, *n* = 7 vs. HSD2BKO 74.28 ± 7.15 nmol 11DHC/mg/h, *n* = 6), the main site of adult expression, where 11β-HSD2 determines aldosterone-selective effects on renal salt-water physiology.

In the adult mouse brain, 11β-HSD2 expression is limited to the nucleus of the tractus solitarius (NTS; Holmes et al., 2006b), where the enzyme might influence blood pressure and electrolyte/fluid volume balance. However, no differences were observed between CON and HSD2BKO littermates in mean arterial blood pressure (CON: 81.2 ± 3.4 mmHg, *n* = 7; HSD2BKO: 74.1 ± 4.5 mmHg, *n* = 7), urinary sodium excretion (CON: 0.231 ± 0.067 μmol/min, *n* = 7; HSD2BKO: 0.319 ± 0.067 μmol/min, *n* = 7), urinary sodium:potassium ratio (CON:1.831 ± 0.383, *n* = 6; HSD2BKO 2.627 ± 0.7037, *n* = 7) or haematocrit (CON: 42.7 ± 0.6%; HSD2BKO: 41.2 ± 1.7%, *n* = 7). These data indicate that adult kidney function, blood pressure and volume homeostasis are not affected by deletion of 11β-HSD2 within the fetal or adult brain. Loss of HSD2 in the adult brain (NTS) is not anticipated to affect affective or cognitive behavior, however it is conceivable (but unlikely) that extremely low levels of 11β-HSD2 transcripts elsewhere in the brain may have a biological function. HSD2BKO mice therefore enable determination of whether 11β-HSD2 expression in the fetal brain modulates glucocorticoid programming of behavior in later life, despite normal placental and kidney expression.

### 3.2 Effects of deleting brain 11β-HSD2 on basic phenotype parameters

Deleting 11β-HSD2 from the brain had no impact on Mendelian ratio, placental or fetal weights at E15.5 and E17.5, nor birthweight or pup survival, when compared to littermate controls (data not shown). These data are indicative of normal growth of the placenta and fetus, which is in contrast to the growth retardation observed in the global 11β-HSD2 knockout model – similarly compared to littermates (Holmes et al., 2006a; Wyrwoll et al., 2009). Body weight was unaltered between CON and HSD2BKO offspring until after weaning when a reduction in body weight of ∼10% was observed in the HSD2BKO offspring (Figure S1). Further investigations revealed that this growth trajectory closely followed that of NestinCre animals (Figure S1), therefore the differences in body weight are attributable to the presence of Cre in the HSD2BKO offspring as opposed to central removal of 11β-HSD2. Neurodevelopment markers and cerebellar morphology were unaltered in HSD2BKO in comparison to controls (Table S2); which is also in contrast to 11β-HSD2^−/−^ mice (Holmes et al., 2006b). Locomotor activity and balance (rotarod performance) were similar across littermate genotypes (latency to fall in CON mice: 110.23 ± 34.5 s vs. HSD2BKO: 105.17 ± 19.84 s), indicating no motor impairment that could confound behavioral tests. There were no differences in food or water intake between CON and HSD2BKO mice.

### 3.3 HSD2BKO mice exhibit transiently increased fetal brain corticosterone levels

To determine whether the reduction in 11β-HSD2 activity in the fetal brain has a functional consequence, we measured brain corticosterone levels, which we hypothesised would be increased due to loss of a local glucocorticoid-inactivating pathway in the HSD2BKO mice. Corticosterone in fetal brain was increased at E15.5 in HSD2BKO compared to CON, at a time when fetal brain 11β-HSD2 expression is normally high (2-way ANOVA shows a significant interaction of genotype and age, *F*(1,27) = 45.59, *p* < 0.05; post hoc *t*-test comparing genotypes at E15.5 *p* < 0.05; Fig. 1B). Importantly, no difference in brain corticosterone concentrations was observed at E17.5 (Fig. 1B), an age when most of brain 11β-HSD2 expression has been repressed (Brown et al., 1996a; Diaz et al., 1998). There was no effect of deletion of 11β-HSD2 on brain expression of 11β-HSD1 activity (E17.5; conversion of 11-dehydrocorticosterone to corticosterone; CON: 0.113 ± 0.035 nmol/mg/h, *n* = 5; VS HSD2BKO: 0.110 ± 0.045 nmol/mg/h, *n* = 6)

### 3.4 HSD2BKO mice exhibit no changes in HPA axis function

Glucocorticoid programming in several models, such as pre- and post-natal stress, results in hyperactivity of the HPA axis in development and throughout life (Harris and Seckl, 2011). This phenomenon was not observed in global 11β-HSD2^−/−^ adult mice, except for decreased adrenal weights, most likely resulting from a prolonged half-life of corticosterone due to decreased catabolism largely in the kidneys, leading to the need for less HPA axis drive to maintain normal plasma glucocorticoid levels. Compatible with this notion, brain-specific deletion of 11β-HSD2 had no effect on adrenal weight (Table 1).

Expression of genes involved in regulation of the HPA axis and glucocorticoid signaling (corticotrophin releasing hormone (*Crh*), *Nr3c1* and *Nr3c2*) in the hypothalamus and hippocampus were unchanged in HSD2BKO compared to CON (Table 1). Plasma corticosterone levels were similar between genotypes at E17.5 and in the adult offspring both basally and in response to restraint stress (Table 1). Thus, HPA axis parameters were not altered in the fetus or adult by 11β-HSD2 deletion in the brain.

### 3.5 Programmed behavioral phenotype in HSD2BKO

#### 3.5.1 HSD2BKO mice do not exhibit anxiety-like behaviors

**Figure 2:**
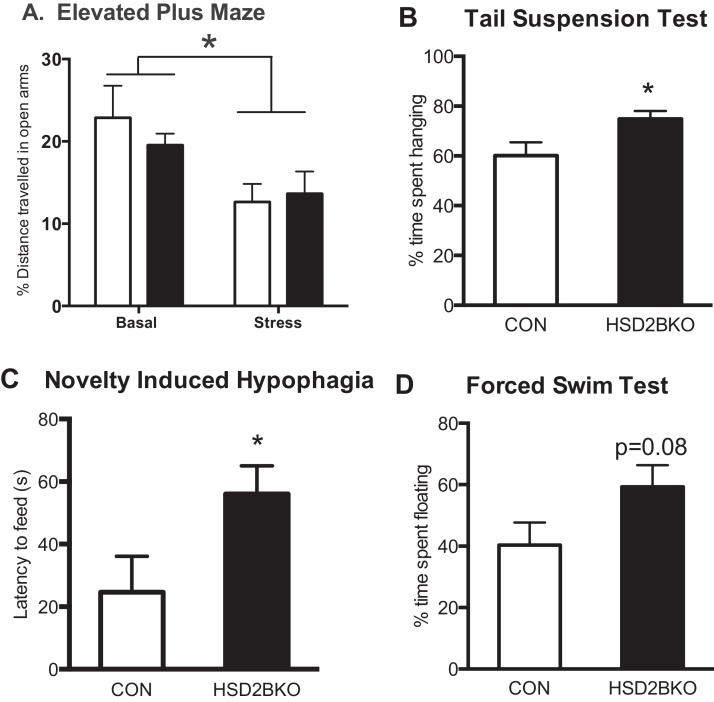
Anxiety-like and depressive-like behaviors in HSD2BKO mice compared to littermate controls (CON). (A) Exploration of the open arms of the Elevated-plus maze (EPM) did not differ between genotypes although restraint stress prior to testing did reduce exploration of the open arm (*p < 0.05, Two-way ANOVA *F*(1,63) = 6.07, *n* = 10–23). HSD2BKO exhibited increased depressive-like behavior in the B. Tail suspension test (TST; *t*-test *t* = 2.25) and (C) Novelty Induced hypophagia test (NIH; *t*-test *t* = 2.14), with a trend for the HSD2BKO mice to spend more time floating in the Forced swim test (FST; D). Values are mean ± SEM, *n* = 11 (CON) and 9 (HSD2BKO). *p < 0.05 compared to CON.

As the offspring of maternal stress or 11β-HSD2^−/−^ mice exhibit increased anxiety, we determined if 11β-HSD2 in the brain, rather than the placenta, protects against programming of affect. Exploration (%distance travelled on the open arms/total distance) of the elevated plus maze (EPM) did not differ between CON and HSD2BKO littermates under normal conditions (Fig. 2A). Similarly, no effect of genotype was observed on total distance travelled throughout the maze or the time spent in the anxiogenic open arms (data not shown). Restraint stress decreased exploration of the open arm of EPM (Fig. 2A) but this was also unaltered by genotype.

Total movement within the open field and exploration of the more anxiogenic inner zones, did not differ between CON and HSD2BKO animals (distance travelled in inner zone by CON mice: 13.14 ± 8.31 cm; *n* = 12 vs. HSD2BKO: 13.02 ± 7.60 cm; *n* = 11. Time spent in the inner zone by CON mice: 2.32 ± 0.47 s, *n* = 12 vs. HSD2BKO: 1.93 ± 0.56 s, *n* = 11). Hence removing 11β-HSD2 from the brain during development did not impact on anxiety behavior. In simultaneous experiments, the increased anxiety-like phenotype of the global 11β-HSD2^−/−^ was confirmed (data not shown). These data suggest that 11β-HSD2 in the placenta (or elsewhere in the fetus), rather than the brain, determines programming of anxiety-like behaviors.

#### 3.5.2 HSD2BKO mice exhibit ‘depressive-like’ behavior

We carried out three tests of depressive-like behavior: tail suspension, novelty-induced hypophagia (NIH) and forced-swim tests. In the tail suspension test, HSD2BKO animals spent a greater proportion of time hanging, as opposed to struggling, than CON littermates (Fig. 2B), indicating a more depressive-like behavioral phenotype. In the NIH test, HSD2BKO mice exhibited an increased latency to feed on the test day in comparison to CON littermates (Fig. 2C) also indicative of increased depressive-like behavior. There were no differences in the training response or the distance travelled in the novel cage between genotypes. The forced swim test revealed no significant differences in behavior between the genotypes (Fig. 2D) though there was a trend for HSD2BKO to float more (a depressive-like behavior). Thus, HSD2BKO mice exhibit more depressive-like behaviors than littermate controls.

#### 3.5.3 Memory is impaired in HSD2BKO mice

**Figure 3:**
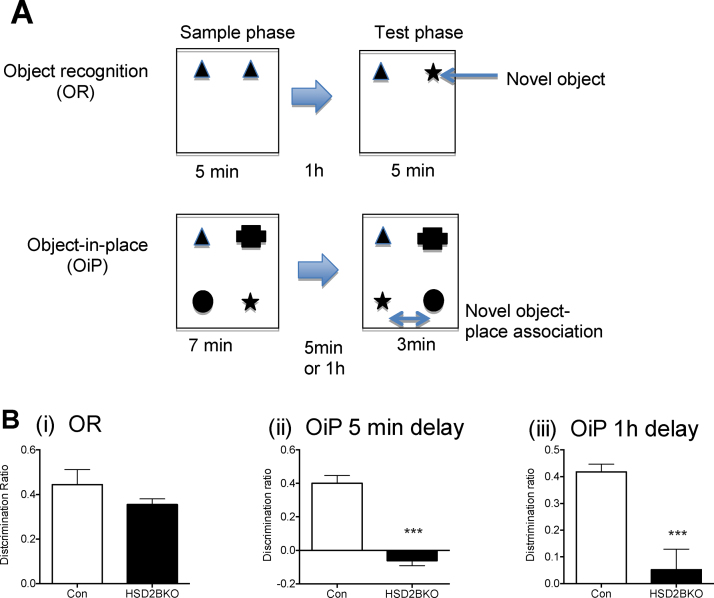
Cognitive behaviors in HSD2BKO compared to CON mice. (A) A cartoon describes the object recognition (OR) test where sample phase enables the mouse to investigate two objects followed by a test phase where one object is changed to a new one, the time the mouse investigates both the known and novel objects is measured and the discrimination ratio is then calculated. The higher the ratio the more the mouse has recognised the object is novel. The second cartoon describes the Object-in-Place (OiP) task. This test involves 4 objects in the sample phase and in the test phase two of the objects have switched place. (B) (i) Object recognition task in C0N and HSD2BKO revealed in performance in this task between genotypes. However in the more demanding task of OiP, while the CON mice can discriminate when objects have been moved to a different place, the HSD2BKO mice are unable to do this irrespective of whether they have had a delay of 5 min (ii) or 1 h (iii) between sample and test phases. Values are mean ± SEM, *n* = 8–9. ***p < 0.001 compared to CON.

Adverse early-life environments are also associated with programming of cognitive impairments. Hence we explored whether deletion of 11β-HSD2 within the brain alters cognition in object recognition (OR) and object-in-place (OiP) tasks, described in Fig. 3A. HSD2BKO mice did not display any deficits compared to CON in the OR task (Fig. 3B(i)) but overt cognitive defects emerged when mice were tested in the more complex, demanding OiP test. With both a 5 min or a 1 h delay between sample and test phase, the HSD2BKO mice failed to discriminate between the 2 objects that had swapped positions and the two that had remained in the same positions as in the sample phase (the discrimination ratio was not different from 0; *p* = 0.084 and *p* = 0.51 for 5 min and 1 h respectively), while control littermates showed discrimination at both time delays by investigating the moved objects longer than the objects that were in their original position. Furthermore, there was a significant reduction in memory in the HSD2BKO compared to CON mice in this test at both 5 min and 1 h delay (DR for 5 min delay was significantly different between genotypes, *t*-test *t* = 8.427, df = 14, *p* = 0.001; DR for 1 h delay, *t*-test *t* = 4.484, df = 14, *p* = 0.001. *p* < 0.0001; Fig. 3B(ii and iii)).

**Figure 4:**
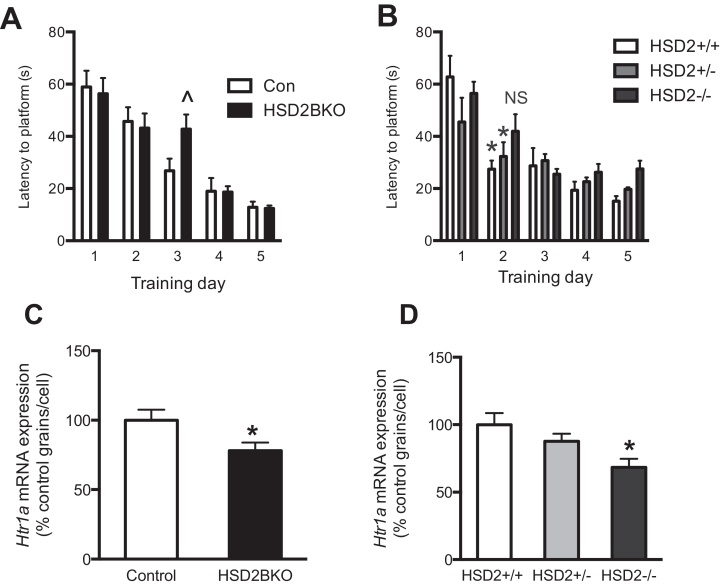
Watermaze learning and hippocampal expression of 5-HT_1A_ receptor mRNA in HSD2BKO and global 11β-HSD2 KO mice (11β-HSD2^−/−^). (A) During the watermaze training period there is a significant deficiency in performance on day 3 of training in the HSD2BKO compared to CON (^*p* < 0.05 compared to CON day 3; *n* = 7–8). (B) 11β-HSD2^+/+^ and ^+/−^ mice exhibit a significant improvement in performance of the task between day 1 and 2, whereas 11β-HSD2^−/−^, do not exhibit an improvement (*n* = 8–10). Hippocampal *Htr1a* mRNA expression in the CA1 field of HSD2BKO (C) and heterozygous-bred global knockouts of 11β-HSD2 (D) as assessed by in situ hybridisation histochemistry was significantly decreased in comparison to controls (CON: *n* = 5 vs. HSD2BKO: *n* = 6; *p < 0.05; 11β-HSD2^+/+^, 11β-HSD2^+/−^ vs. 11β-HSD2^−/−^: *n* = 6 in all cases, *p < 0.05, one-way ANOVA). Values are mean ± SEM; *n* = 5–6.

In a spatial reference memory task conducted in the Morris water maze, HSD2BKO mice did not differ from CON mice in the swim speed (CON: 23.5 ± 1.5 cm/s, *n* = 10; HSD2BKO 25.2 ± 0.9 cm/s, *n* = 10), visible platform trial (latency to platform: CON: 21.6 ± 5.2 s, *n* = 10; HSD2BKO 21.7 ± 5.0 s, *n* = 10) or probe trial (latency to platform: CON: 17.5 ± 6.9 s, *n* = 10; HSD2BKO 14.7 ± 3.8 s, *n* = 10). They did however exhibit small differences in spatial learning at specific times during the training phase, with HSD2BKO mice demonstrating a delay in locating the platform on Day 3 (Fig. 4A). Similar delays in task learning were also seen in global 11β-HSD2^−/−^ mice between Day 1 and 2 (Fig. 4B). Hence, only minor deficiencies in learning this task was observed in mice lacking 11β-HSD2 in the brain or globally and therefore only very complex demanding tasks are able to reveal the cognitive deficit, at least in young mice.

### 3.6 Neurotransmitter levels are unaltered but 5-HT_1A_ receptor mRNA expression is decreased in the hippocampus of HSD2BKO mice

Levels of neurotransmitters and their receptor mRNAs which underpin affective behaviors were measured in selected brain areas. No differences were observed in serotonin, dopamine or their metabolites between CON and HSD2BKO mice in any brain region investigated (Table S3), contrary to observations in global 11β-HSD2^−/−^ mice. Strikingly, 5-HT_1A_ receptor (*Htr1a*) mRNA levels were reduced in the CA1 field of the hippocampus in HSD2BKO mice (*t*-test *t* = 2.329, df = 9, *p* = 0.0457; Fig. 4C). Representative autoradiographs of *Htr1a* expression in both genotypes are shown in Figure S2. A similar down-regulation of *Htr1a* mRNA was observed in the same subfield of the hippocampus of global 11β-HSD2^−/−^ compared to HSD2^+/+^ littermates (1-way ANOVA *F*(2,15) = 5.234, *p* = 0.0189; Tukey's post hoc test *p* = 0.050; Fig. 4D). No changes between genotypes were observed in mRNA levels of *Tph2*, *Slc6a4*, *Drd2*, or *Slc6a3* in any brain region.

## 4 Discussion

We show that absence of 11β-HSD2 solely in the developing brain results in a specific, delineated stratification of the phenotype observed in complete feto-placental 11β-HSD2 deficiency. Thus, increased depressive-like behaviors and cognitive impairments in adulthood are related to loss of 11β-HSD2 in the fetal CNS, whilst the anxiety component of global 11β-HSD2 deficiency and many other programming models, is not observed. This allows dissection of potential differential mechanisms underpinning programming of important brain functions.

The fetal growth restriction resulting from reduced feto-placental 11β-HSD2 expression and hence placental dysfunction, observed in most models of prenatal stress/glucocorticoid overexposure (including global 11β-HSD2^−/−^ mice; (Hewitt et al., 2006; Lucassen et al., 2009; Wyrwoll et al., 2009) and with maternal nutritional restriction (Langley-Evans et al., 1996)), is absent in HSD2BKO. This suggests that programming of the fetus in this model is caused by exposure to fetal rather than maternal corticosterone and is a direct action on the brain. This suggests that HSD2BKO contributes to programming of depressive-like behavior but not growth restriction, whereas feto-placental 11β-HSD2 expression contributes to both. In keeping with this, fetal brain levels of corticosterone are elevated in HSD2BKO mice, but only at time-points when 11β-HSD2 expression is high. The fetal HPA axis is not overtly altered by removal of 11β-HSD2 in the fetal CNS (unaltered *Crh Nr3c1*, *Nr3c2* mRNA levels in the fetal PVN and hippocampus, normal plasma corticosterone in the fetus). These data indicate that glucocorticoids accumulate selectively in the fetal brain with loss of local 11β-HSD2. Factors that disrupt the complex patterns (temporal and spatial) of 11β-HSD2 inactivation during brain development would therefore be of substantial interest.

Exposure to increased glucocorticoids during fetal development can affect working and reference memory, impair long term potentiation and favour long term depression (Kamphuis et al., 2003; Yaka et al., 2007). The occurrence of memory deficits is dependent on the magnitude and duration of exposure to glucocorticoids combined with the stage of development (Cannizzaro et al., 2006; Lemaire et al., 2000; Yaka et al., 2007). Here, simple hippocampus-encoded reference memory tasks were minimally affected in HSD2BKO mice, yet marked cognitive defects were revealed using the more complex object-in-place task, which integrates information related to object placement and object recognition requiring contributions of multiple regions including the hippocampus, perirhinal cortex and the medial prefrontal cortex (Barker et al., 2007; Barker and Warburton, 2011). Whether the cognitive deficits observed in HSD2BKO mice is driven by a single brain locus particularly sensitive to developmental glucocorticoid actions or is the result of the increased task challenge on a generally dysfunctional CNS remains unclear. Such effects fit with the cognitive defects observed in the children of mothers consuming larger amounts of licorice in pregnancy (Raikkonen et al., 2009). All of the behavioral testing was done in the early light phase, while circadian effects on behavior are at present unknown.

Many early-life manipulations, for example prenatal stress, are associated with life-long overactivity of the HPA axis hence generating the hypothesis that elevated glucocorticoid levels may cause the observed phenotype resulting from glucocorticoid programming. However programming of affective behaviors was not associated with major modifications of HPA axis function in either 11β-HSD2^−/−^ (Holmes et al., 2006a) or HSD2BKO mice. Although, HPA axis changes are correlated with programming induced by prenatal stress across many species, only very limited data, using stress timepoints very early in gestation, has reported such changes in the mouse to date (Mueller and Bale, 2008). It is unknown if the mouse is relatively resistant to HPA axis programming or whether further factors released in response to stress are required to generate these effects. However, HPA axis changes per se are not likely to be responsible for generation of the affective and cognitive phenotypes, at least with 11β-HSD2 deficiency and perhaps other challenges.

This leaves the interesting question of why depressive, but not anxiety, behaviors are programmed in the HSD2BKO. The incomplete loss of 11β-HSD2 in the HSD2BKO brain may be involved. However, we think this is unlikely as although the HSD2BKO has ∼25% 11β-HSD2 activity in the brain compared to controls, the mRNA levels are negligible (<4%). Indeed, in a prenatal stress model the anxiety and depressive-like behaviors appeared to be due to two unrelated psychopathologies (Marrocco et al., 2014). This behavioral selectivity may therefore be generated by indirect signalling of factors such as insulin-like growth factor 2 (which facilitates placental response to fetal growth demand (Constancia et al., 2005)) from the growth restricted placenta and/or fetus, or the timing (differentially affecting neural circuitry) and magnitude of the glucocorticoid exposure to the fetal brain. Whatever the basis, separating the programming of depressive-like and anxiety-like behaviors is of substantial interest.

Dysfunction of monoamine signalling occurs in affective disorders and follows glucocorticoid exposure in early life (Gartside et al., 2003; Goodfellow et al., 2009; Miyagawa et al., 2011; Muneoka et al., 1997; Van den Hove et al., 2006). *Htr1a* mRNA expression was altered in both global and HSD2BKO models, with a consistent downregulation of expression in the CA1 field of the hippocampus. This finding is of key interest, as altered serotonin responsiveness of CA1 hippocampal neurons as a consequence of stress has been implicated in mood disorders (Krugers et al., 2010). Indeed, reduced 5HT_1A_ receptor binding is seen in the brain in depression in humans (Bhagwagar et al., 2004; Sargent et al., 2000) and partial 5-HT_1A_ receptor agonists are antidepressants (Blier and Ward, 2003). Decreased binding of hippocampal 5-HT_1A_ receptors have been found in models of early life stress (Franklin et al., 2011) while chronic stress alters the responsiveness of 5-HT_1A_ (Karten et al., 1999; van Riel et al., 2003). Environmental enrichment which reverses programmed affective and cognitive behaviors promotes increased expression of *Htr1a* (Rasmuson et al., 1998). This system which underpins depressive-like phenotypes appears particularly sensitive to glucocorticoid programming directly in the fetal brain.

There has been considerable research into the regulation of 11β-HSD2 expression in placenta (environmental factors such as proinflammatory cytokines, glucocorticoids, toxins, and epigenetic mark changes reported) as this was thought to be the main site of expression relevant in glucocorticoid programming. We have now shown that the brain 11β-HSD2 is important too, pressing the need for research into fetal brain regulation of 11β-HSD2. Some data suggest epigenetic changes occur in the 11β-HSD2 promoter in fetal hypothalamus in response to maternal stress (Jensen Pena et al., 2012) and fetal brain 11β-HSD2 mRNA responds to alterations in maternal nutrition in pigs (Kanitz et al., 2012). However, more studies are needed to determine factors that regulate 11β-HSD2 expression in the brain and hence the susceptibility to psychiatric diseases.

## 5 Conclusions

In sum, we show that fetal brain programming of specific functions (depressive-like behavior, cognition) is powerfully influenced by local glucocorticoid levels determined by fetal brain 11β-HSD2, whilst programming of anxiety-like behavior and the HPA axis are not a direct effect of local 11β-HSD2 control of glucocorticoid access to the brain. These data illustrate exquisite and complex control of glucocorticoid action upon the developing CNS and imply multiple targets in the control of the trajectory of development of behavior, cognition and neuroendocrine function during normal and disordered development. Understanding the control of fetal brain 11β-HSD2 is of substantial interest.

## Role of the funding source

We acknowledge Wellcome Trust project grant (WT079009) and EU FP7 collaborative grant Developmental origins of healthy and unhealthy ageing (DORIAN) to MCH and JRS. The work was supported by a PhD studentship (SC) from The University of Edinburgh Centre for Cognitive Ageing and Cognitive Epidemiology, part of the cross council Lifelong Health and Wellbeing Initiative (G0700704). Funding from the Biotechnology and Biological Sciences Research Council (BBSRC), Engineering and Physical Sciences Research Council (EPSRC), Economic and Social Research Council (ESRC), and Medical Research Council (MRC) is gratefully acknowledged.

None of the funders were involved in study design; in the collection, analysis and interpretation of data; in the writing of the report; and in the decision to submit the article for publication.

## Conflict of interest

None declared.

## Figures and Tables

**Table 1:** The effect of HSD2BKO on HPA axis parameters.

	CON	HSD2BKO
Adrenal size: body weight (adult)	5.9 ± 0.9 × 10^−4^ (15)	4.9 ± 0.9 × 10^−4^ (18)

**Brain gene expression** (*n* = 5–8)		
*Crh* (E15.5; PVN)(ROD)	0.336 ± 0.01	0.320 ± 0.026
*Crh* (E17.5; PVN)(ROD)	0.274 ± 0.0181	0.252 ± 0.016
Nr3c2 (E15.5; HC)(ROD)	ND	ND
*Nr3c2* (E17.5; HC)(ROD)	0.26 ± 0.02	0.26 ± 0.01
*Nr3c2* (Adult; HC) (norm to HPRT)	0.868 ± 0.077	0.818 ± 0.061
*Nr3c1* (Adult; HC)(norm to HPRT)	0.733 ± 0.170	0.744 ± 0.055
*Hsd11b1* (E12.5; norm to HPRT)	0.172 ± 0.033	0.155 ± 0.021
*Hsd11b1* (E17.5; norm to HPRT)	0.188 ± 0.028	0.148 ± 0.022

**Plasma corticosterone (nmol/l)**		
Fetal (E17.5)	31.2 ± 2.1	28.1 ± 2.0

**Adult**	(10)	(9)
AM	35.3 ± 4.8	34.3 ± 6.7
PM	196.5 ± 16.6	179.9 ± 22.9

**Restraint stress corticosterone levels (nmol/l)**	(12)	(12)
0 min restraint stress	33.8 ± 4.7	35.9 ± 5.3
30 min restraint stress	622.9 ± 87.4	661.0 ± 98.1
1 h post restraint stress	155.7 ± 27.8	181.9 ± 26.8

There were no significant differences between CON and HSD2BKO in any of the parameters of the HPA axis. Values are mean ± SEM, (N). PVN, nucleus; HC, hippocampus; ROD, relative optical density; ND, not detectable; norm HPRT, genes expression normalised to housekeeping gene, hypoxanthine phosphoribosyltransferase. Fetal Nr3c1 could not be quantitated due to very high neuroepthelia expression masking hippocampal expression.

## References

[bib0005] Barker G.R., Bird F., Alexander V., Warburton E.C. (2007). Recognition memory for objects, place, and temporal order: a disconnection analysis of the role of the medial prefrontal cortex and perirhinal cortex. J. Neurosci..

[bib0010] Barker G.R., Warburton E.C. (2011). When is the hippocampus involved in recognition memory?. J. Neurosci..

[bib0015] Benediktsson R., Calder A.A., Edwards C.R., Seckl J.R. (1997). Placental 11 beta-hydroxysteroid dehydrogenase: a key regulator of fetal glucocorticoid exposure. Clin. Endocrinol. (Oxf.).

[bib0020] Bhagwagar Z., Rabiner E.A., Sargent P.A., Grasby P.M., Cowen P.J. (2004). Persistent reduction in brain serotonin1A receptor binding in recovered depressed men measured by positron emission tomography with [11C]WAY-100635. Mol. Psychiatry.

[bib0025] Blier P., Ward N.M. (2003). Is there a role for 5-HT1A agonists in the treatment of depression?. Biol. Psychiatry.

[bib0030] Brown R.W., Chapman K.E., Edwards C.R., Seckl J.R. (1993). Human placental 11 beta-hydroxysteroid dehydrogenase: evidence for and partial purification of a distinct NAD-dependent isoform. Endocrinology.

[bib0035] Brown R.W., Diaz R., Robson A.C., Kotelevtsev Y.V., Mullins J.J., Kaufman M.H., Seckl J.R. (1996). The ontogeny of 11 beta-hydroxysteroid dehydrogenase type 2 and mineralocorticoid receptor gene expression reveal intricate control of glucocorticoid action in development. Endocrinology.

[bib0040] Brown R.W., Kotolevtsev Y., Leckie C., Lindsay R.S., Lyons V., Murad P., Mullins J.J., Chapman K.E., Edwards C.R.W., Seckl J.R. (1996). Isolation and cloning of human placental 11β-hydroxysteroid dehydrogenase-2 cDNA. Biochem. J.

[bib0045] Burton P.J., Smith R.E., Krozowski Z.S., Waddell B.J. (1996). Zonal distribution of 11 beta-hydroxysteroid dehydrogenase types 1 and 2 messenger ribonucleic acid expression in the rat placenta and decidua during late pregnancy. Biol. Reprod..

[bib0050] Cannizzaro C., Plescia F., Martire M., Gagliano M., Cannizzaro G., Mantia G., Cannizzaro E. (2006). Single, intense prenatal stress decreases emotionality and enhances learning performance in the adolescent rat offspring: interaction with a brief, daily maternal separation. Behav. Brain Res..

[bib0055] Constancia M., Angiolini E., Sandovici I., Smith P., Smith R., Kelsey G., Dean W., Ferguson-Smith A., Sibley C.P., Reik W., Fowden A. (2005). Adaptation of nutrient supply to fetal demand in the mouse involves interaction between the Igf2 gene and placental transporter systems. Proc. Natl. Acad. Sci. U. S. A..

[bib0060] Cost K.T., Lobell T.D., Williams-Yee Z.N., Henderson S., Dohanich G. (2014). The effects of pregnancy, lactation, and primiparity on object-in-place memory of female rats. Horm. Behav..

[bib0065] Cottrell E.C., Holmes M.C., Livingstone D.E., Kenyon C.J., Seckl J.R. (2012). Reconciling the nutritional and glucocorticoid hypotheses of fetal programming. FASEB J..

[bib0070] Cottrell E.C., Seckl J.R. (2009). Prenatal stress, glucocorticoids and the programming of adult disease. Front. Behav. Neurosci..

[bib0075] Cottrell E.C., Seckl J.R., Holmes M.C., Wyrwoll C.S. (2014). Foetal and placental 11beta-HSD2: a hub for developmental programming. Acta Physiol. (Oxf).

[bib0080] Diaz R., Brown R.W., Seckl J.R. (1998). Distinct ontogeny of glucocorticoid and mineralocorticoid receptor and 11beta-hydroxysteroid dehydrogenase types I and II mRNAs in the fetal rat brain suggest a complex control of glucocorticoid actions. J. Neurosci..

[bib0085] Franklin T.B., Linder N., Russig H., Thony B., Mansuy I.M. (2011). Influence of early stress on social abilities and serotonergic functions across generations in mice. PLoS ONE.

[bib0090] Gartside S.E., Johnson D.A., Leitch M.M., Troakes C., Ingram C.D. (2003). Early life adversity programs changes in central 5-HT neuronal function in adulthood. Eur. J. Neurosci..

[bib0095] Goodfellow N.M., Benekareddy M., Vaidya V.A., Lambe E.K. (2009). Layer II/III of the prefrontal cortex: inhibition by the serotonin 5-HT1A receptor in development and stress. J. Neurosci..

[bib0100] Harris A., Seckl J. (2011). Glucocorticoids, prenatal stress and the programming of disease. Horm. Behav..

[bib0105] Harris H.J., Kotelevtsev Y., Mullins J.J., Seckl J.R., Holmes M.C. (2001). Intracellular regeneration of glucocorticoids by 11beta-hydroxysteroid dehydrogenase (11beta-HSD)-1 plays a key role in regulation of the hypothalamic-pituitary-adrenal axis: analysis of 11beta-HSD-1-deficient mice. Endocrinology.

[bib0110] Hewitt D.P., Mark P.J., Waddell B.J. (2006). Glucocorticoids prevent the normal increase in placental vascular endothelial growth factor expression and placental vascularity during late pregnancy in the rat. Endocrinology.

[bib0115] Holmes M.C., Abrahamsen C.T., French K.L., Paterson J.M., Mullins J.J., Seckl J.R. (2006). The mother or the fetus? 11beta-hydroxysteroid dehydrogenase type 2 null mice provide evidence for direct fetal programming of behavior by endogenous glucocorticoids. J. Neurosci..

[bib0120] Holmes M.C., French K.L., Seckl J.R. (1997). Dysregulation of diurnal rhythms of serotonin 5-HT2C and corticosteroid receptor gene expression in the hippocampus with food restriction and glucocorticoids. J. Neurosci..

[bib0125] Holmes M.C., Sangra M., French K.L., Whittle I.R., Paterson J., Mullins J.J., Seckl J.R. (2006). 11β-Hydroxysteroid dehydrogenase type 2 protects the neonatal cerebellum from deleterious effects of glucocorticoids. Neuroscience.

[bib0130] Hunter R.W., Craigie E., Homer N.Z., Mullins J.J., Bailey M.A. (2014). Acute inhibition of NCC does not activate distal electrogenic Na+ reabsorption or kaliuresis. Am. J. Physiol. Renal Physiol..

[bib0135] Jensen Pena C., Monk C., Champagne F.A. (2012). Epigenetic effects of prenatal stress on 11beta-hydroxysteroid dehydrogenase-2 in the placenta and fetal brain. PLoS ONE.

[bib0140] Kamphuis P.J., Gardoni F., Kamal A., Croiset G., Bakker J.M., Cattabeni F., Gispen W.H., van Bel F., Di Luca M., Wiegant V.M. (2003). Long-lasting effects of neonatal dexamethasone treatment on spatial learning and hippocampal synaptic plasticity. Involvement of the NMDA receptor complex. FASEB J..

[bib0145] Kanitz E., Otten W., Tuchscherer M., Grabner M., Brussow K.P., Rehfeldt C., Metges C.C. (2012). High and low protein ratio carbohydrate dietary ratios during gestation alter maternal-fetal cortisol regulation in pigs. PLoS ONE.

[bib0150] Karten Y.J., Nair S.M., van Essen L., Sibug R., Joels M. (1999). Long-term exposure to high corticosterone levels attenuates serotonin responses in rat hippocampal CA1 neurons. Proc. Natl. Acad. Sci. U. S. A..

[bib0155] Khashan A.S., McNamee R., Henriksen T.B., Pedersen M.G., Kenny L.C., Abel K.M., Mortensen P.B. (2011). Risk of affective disorders following prenatal exposure to severe life events: a Danish population-based cohort study. J. Psychiatr. Res..

[bib0160] Krugers H.J., Lucassen P.J., Karst H., Joels M. (2010). Chronic stress effects on hippocampal structure and synaptic function: relevance for depression and normalization by anti-glucocorticoid treatment. Front. Synaptic Neurosci..

[bib0165] Langley-Evans S.C., Phillips G.J., Benediktsson R., Gardner D.S., Edwards C.R., Jackson A.A., Seckl J.R. (1996). Protein intake in pregnancy, placental glucocorticoid metabolism and the programming of hypertension in the rat. Placenta.

[bib0170] Lemaire V., Koehl M., Le Moal M., Abrous D.N. (2000). Prenatal stress produces learning deficits associated with an inhibition of neurogenesis in the hippocampus. Proc. Natl. Acad. Sci. U.S.A..

[bib0175] Lucassen P.J., Bosch O.J., Jousma E., Kromer S.A., Andrew R., Seckl J.R., Neumann I.D. (2009). Prenatal stress reduces postnatal neurogenesis in rats selectively bred for high, but not low, anxiety: possible key role of placental 11beta-hydroxysteroid dehydrogenase type 2. Eur. J. Neurosci..

[bib0180] Mairesse J., Lesage J., Breton C., Breant B., Hahn T., Darnaudery M., Dickson S.L., Seckl J., Blondeau B., Vieau D., Maccari S., Viltart O. (2007). Maternal stress alters endocrine function of the feto-placental unit in rats. Am. J. Physiol. Endocrinol. Metab..

[bib0185] Marrocco J., Reynaert M.L., Gatta E., Gabriel C., Mocaer E., Di Prisco S., Merega E., Pittaluga A., Nicoletti F., Maccari S., Morley-Fletcher S., Mairesse J. (2014). The effects of antidepressant treatment in prenatally stressed rats support the glutamatergic hypothesis of stress-related disorders. J. Neurosci..

[bib0190] Miyagawa K., Tsuji M., Fujimori K., Saito Y., Takeda H. (2011). Prenatal stress induces anxiety-like behavior together with the disruption of central serotonin neurons in mice. Neurosci. Res..

[bib0195] Mombereau C., Gur T.L., Onksen J., Blendy J.A. (2010). Differential effects of acute and repeated citalopram in mouse models of anxiety and depression. Int. J. Neuropsychopharmacol..

[bib0200] Mueller B.R., Bale T.L. (2008). Sex-specific programming of offspring emotionality after stress early in pregnancy. J. Neurosci..

[bib0205] Muneoka K., Mikuni M., Ogawa T., Kitera K., Kamei K., Takigawa M., Takahashi K. (1997). Prenatal dexamethasone exposure alters brain monoamine metabolism and adrenocortical response in rat offspring. Am. J. Physiol..

[bib0210] O’Donnell K.J., Bugge Jensen A., Freeman L., Khalife N., O’Connor T.G., Glover V. (2012). Maternal prenatal anxiety and downregulation of placental 11beta-HSD2. Psychoneuroendocrinology.

[bib0215] Pesonen A.K., Raikkonen K., Lano A., Peltoniemi O., Hallman M., Kari M.A. (2009). Antenatal betamethasone and fetal growth in prematurely born children: implications for temperament traits at the age of 2 years. Pediatrics.

[bib0220] Pryce C.R., Aubert Y., Maier C., Pearce P.C., Fuchs E. (2011). The developmental impact of prenatal stress, prenatal dexamethasone and postnatal social stress on physiology, behaviour and neuroanatomy of primate offspring: studies in rhesus macaque and common marmoset. Psychopharmacology (Berl).

[bib0225] Raikkonen K., Pesonen A.K., Heinonen K., Lahti J., Komsi N., Eriksson J.G., Seckl J.R., Jarvenpaa A.L., Strandberg T.E. (2009). Maternal licorice consumption and detrimental cognitive and psychiatric outcomes in children. Am. J. Epidemiol..

[bib0230] Raikkonen K., Seckl J.R., Heinonen K., Pyhala R., Feldt K., Jones A., Pesonen A.K., Phillips D.I., Lahti J., Jarvenpaa A.L., Eriksson J.G., Matthews K.A., Strandberg T.E., Kajantie E. (2010). Maternal prenatal licorice consumption alters hypothalamic-pituitary-adrenocortical axis function in children. Psychoneuroendocrinology.

[bib0235] Rasmuson S., Olsson T., Henriksson B.G., Kelly P.A., Holmes M.C., Seckl J.R., Mohammed A.H. (1998). Environmental enrichment selectively increases 5-HT1A receptor mRNA expression and binding in the rat hippocampus. Brain Res. Mol. Brain Res..

[bib0240] Sargent P.A., Kjaer K.H., Bench C.J., Rabiner E.A., Messa C., Meyer J., Gunn R.N., Grasby P.M., Cowen P.J. (2000). Brain serotonin1A receptor binding measured by positron emission tomography with [11C]WAY-100635: effects of depression and antidepressant treatment. Arch. Gen. Psychiatry.

[bib0245] Steru L., Chermat R., Thierry B., Simon P. (1985). The tail suspension test: a new method for screening antidepressants in mice. Psychopharmacology (Berl).

[bib0250] Trautman P.D., Meyer-Bahlburg H.F.L., Postelnek J., New M.I. (1995). Effects of early prenatal dexamethasone on the cognitive and behavioral development of young children: results of a pilot study. Psychoneuroendocrinology.

[bib0255] Tronche F., Kellendonk C., Kretz O., Gass P., Anlag K., Orban P.C., Bock R., Klein R., Schütz G. (1999). Disruption of the glucocorticoid receptor gene in the nervous system results in reduced anxiety. Nat. Genet..

[bib0260] Van den Hove D.L., Lauder J.M., Scheepens A., Prickaerts J., Blanco C.E., Steinbusch H.W. (2006). Prenatal stress in the rat alters 5-HT1A receptor binding in the ventral hippocampus. Brain Res..

[bib0265] van Riel E., Meijer O.C., Steenbergen P.J., Joels M. (2003). Chronic unpredictable stress causes attenuation of serotonin responses in cornu ammonis 1 pyramidal neurons. Neuroscience.

[bib0270] Weinstock M. (2008). The long-term behavioural consequences of prenatal stress. Neurosci. Biobehav. Rev..

[bib0275] Welberg L.A., Seckl J.R., Holmes M.C. (2000). Inhibition of 11beta-hydroxysteroid dehydrogenase, the foeto-placental barrier to maternal glucocorticoids, permanently programs amygdala GR mRNA expression and anxiety-like behaviour in the offspring. Eur. J. Neurosci..

[bib0280] Wyrwoll C.S., Holmes M.C. (2012). Prenatal excess glucocorticoid exposure and adult affective disorders: a role for serotonergic and catecholamine pathways. Neuroendocrinology.

[bib0285] Wyrwoll C.S., Seckl J.R., Holmes M.C. (2009). Altered placental function of 11beta-hydroxysteroid dehydrogenase 2 knockout mice. Endocrinology.

[bib0290] Yaka R., Salomon S., Matzner H., Weinstock M. (2007). Effect of varied gestational stress on acquisition of spatial memory, hippocampal LTP and synaptic proteins in juvenile male rats. Behav. Brain Res..

[bib0295] Yau J.L., Noble J., Kenyon C.J., Hibberd C., Kotelevtsev Y., Mullins J.J., Seckl J.R. (2001). Lack of tissue glucocorticoid reactivation in 11beta -hydroxysteroid dehydrogenase type 1 knockout mice ameliorates age-related learning impairments. Proc. Natl. Acad. Sci. U. S. A..

[bib0300] Yeh T., Lin Y., Lin H., Huang C., Hsieh W., Lin C., Tsai C. (2004). Outcomes at school age after postnatal dexamethasone therapy for lung disease of prematurity. N. Engl. J. Med..

