# Supplementary material for: Fetal brain 11β-hydroxysteroid dehydrogenase type 2 selectively determines programming of adult depressive-like behaviors and cognitive function, but not anxiety behaviors in male mice
Source: Psychoneuroendocrinology. 2015 Sep;59:59–70. doi: 10.1016/j.psyneuen.2015.05.003 (PMC4510145; doi:10.1016/j.psyneuen.2015.05.003)
Supplement: Supplementary file 1 [file mmc1.docx]

**Supplementary Information (SI)**

**Methods**

*Mice*

11β-HSD2^flx/flx^ mice were produced by Artemis Pharmaceuticals (Cologne, Germany) onto a C57BL/6J background. LoxP sites were placed between exons 2 and 5 of the murine 11β-HSD2 sequence, excision of which has previously been shown to generate a null allele ([Kotelevtsev et al., 1999](#_ENREF_5)). Selective removal of 11β-HSD2 from the developing brain was achieved by crossing 11β-HSD2^flx/flx^ with a Cre mouse line driven by the Nestin promotor ([Tronche et al., 1999](#_ENREF_7)), which is expressed in proliferating CNS cells.

For breeding experimental animals, male Nestin-Cre**.**11βHSD2^flx/flx^ mice were crossed with female 11βHSD2^flx/flx^ mice to produce 11βHSD2^flx/flx^ offspring (CON) and Nestin-Cre**.**11βHSD2^flx/flx^ (HSD2BKO), thus removing 11β-HSD2 from the developing brain. For experiments requiring timed matings to generate fetal tissues, male Nestin-Cre**.**11βHSD2^flx/flx^ mice were added to a cage of 2 CON females at 5pm. The male was removed at 8am the next day and the females checked for vaginal plugs. Other breeding was carried out by set breeding pairs remaining together throughout gestation and postnatally with access to nesting material and nesting domes. Additionally, the behavioural phenotype of C57BL/6J, Nestin-Cre and 11βHSD2^flx/flx^ mice were compared to determine whether the insertion of loxP sites into the 11β-HSD2 gene or the insertion of the Nestin-Cre transgene altered any outcomes and 11β-HSD2^+/+^, 11β-HSD2^+/-^, 11β-HSD2^-/-^ offspring from 11β-HSD2^+/-^ matings were used as comparisons for the HSD2BKO phenotype. The offspring were housed identically. Lights were on from 07:00 to 19:00, temperature maintained at 22°C and animals were fed with fed with standard chow and water *ad libitum.* All experiments were performed blind to genotype on male mice using littermate controls. Offspring were weighed weekly to assess postnatal growth trajectories.

*Tissue collections*

Prenatal tissue collections were taken at E12.5 to assess 11β-HSD2 activity in CON and HSD2BKO fetal and placental tissues. Dams were culled and hysterectomized, with the uterus placed immediately on ice. Fetuses and placentas were weighed, and the fetal head and labyrinth zone of the placenta dissected on ice to prevent 11β-HSD2 activity degradation. Tissue was immediately frozen on dry ice and stored at -80°C until determination of 11β-HSD2 activity ([Brown et al., 1993](#_ENREF_1)). Fetal brains were additionally collected at E15.5 and E17.5 and dissected for gene analyses.

It was previously found that global deletion of 11β-HSD2 caused decreased postnatal cerebellar growth (Holmes 2006) and hence to determine if this was replicated in HSD2BKO, cerebellum were dissected at postnatal day (P) 21. Cerebellum were fixed in 4% paraformaldehyde for 24 h and then stored in 70% ethanol. In adults, left adrenals were removed and placed immediately into 4% paraformaldehyde (Sigma-Aldrich, Poole, UK) for 24 h. Post fixation, adrenals were cleaned of any attached fat by manual dissection. Adrenals were then weighed on a microbalance. Brains were either frozen whole, or dissected and frozen.

*Behavioural testing*

All behavioural tests were performed during the light period between 08:00 and 13:00 h on male mice between 3-4 months of age. Animals were acclimatised to the experimental room overnight before the test. When appropriate, all behavioural tests were recorded using the Limelight^TM^ video tracking system and Actimetrics software (Actimetrics Inc., IL, USA) to enable analysis. No behavioural differences were observed in any behavioural test between Nestin-Cre mice, HSD2^flx/flx^ mice and C57BL/6J mice, indicating that any difference in behaviour between the CON and HSD2BKO are due to depletion of 11β-HSD2 in the brain. Alongside these mice, a cohort of mice with global deletion of 11β-HSD2 (11β-HSD2^-/-^ mice), were tested to compare the behavioural phenotype resulting from brain specific removal of 11β-HSD2 to the well-established model of global 11β-HSD2 removal.

*Novelty-induced hypophagia (NIH)*

To conduct the NIH study, singly housed mice were initially trained to consume chocolate chips from a plastic petri dish in their home cage. Mice received five training sessions (max 15 minutes, twice per day), with latency to eat the chocolate chip recorded. On the sixth day, mice were tested in a novel cage environment. The novel environment differed from the home cage via room location, lighting, smell and lack of bedding. Mice were placed into the novel cage for 5 min and distance travelled and latency to eat were recorded, with a longer latency to eat an indicator of depressive-like behaviour.

*Morris water maze test*

The Morris water maze was used to assess behaviour associated with learning and memory of the Con and HSD2BKO mice. Procedures were followed as detailed by Yau et al. ([2001](#_ENREF_9)). Briefly, mice were initially given a visible probe trial where they were taught to swim to a visible platform in a random location. Subsequent to this, mice were given four training trials per day for five days, from a random start location with a hidden platform in a fixed location. For each training session, mice were given 90 s swim time to mount the platform, after which they were guided there manually. Latency to escape the water is a measure of learning ability. At the end of day five, mice were given a probe trial whereby the platform was removed and the time and direction of swim search was considered to be an indicator of memory retention.

*Object and object-location memory*

The procedure for these tasks were informed by previous publications ([Cost et al., 2014](#_ENREF_2)). Exploration data were collected using a video signal from the overhead camera, which fed into a PC running tracking software (Anymaze, Stoelting, Wood Dale, U.S.A.). Timing was performed manually by the experimenter who observed the behavior of the mouse via the monitor and recorded the amount of time the mouse was engaged in active exploration at each object using key presses or mouse clicks.

Object Recognition: Prior to the beginning of the object recognition test, each animal was handled for 2-3 minutes each day for 5 days and then further exposed to a 5 day habituation period where they were gradually acclimatised to the apparatus as well as the presence of two objects, which were not used in subsequent testing, in the northeast (NE) and northwest (NW) of the box to minimise stress. Object recognition consisted of a sample and a test phase, whereby in the sample phase, each animal was place in the 30cm x 30cm square box in the presence of two identical objects and allowed to explore the area for 5 minutes with exploration at each object recorded. Mice must reach a criteria of 10 seconds at each object to progress the test phase. During each interval, the animal was placed in a ‘holding bucket’ for the 5 minutes. In the test phase, a third copy of the object that was presented in the sample phase was placed in one of the positions as previously encountered and the other object was replaced by a completely novel object. The mouse was then allowed to explore the objects/box for 5 minutes and the time spent actively exploring each object was recorded.

Object in Place: Each mouse was handled 2-3 minutes prior the experiment and habituated to the box as well as the presence of objects in all 4 corners over a 5 day period. The sample phase of the experiment involved the mouse being placed in the box facing the south wall and allowed to explore freely for 7 minutes. Time spent exploring each of the 4 objects was recorded and criteria of 10 second exploration at each object had to be met before being allowed to progress to the next stage. Exploration was defined as being within 2 cm of the object, with whiskers directed at the object. Sitting beside or on the object facing in a different direction was not counted as exploration. During the delay phase the mouse was placed in a ‘holding bucket’ for the 5 minutes or back to the home cage for the 1 hour delay, while the box was prepared for the test phase. For this, two of the objects used in the sample phase remained in the same position whereas the other two swapped locations. The object and locations were counterbalanced between groups. The mouse was then placed back in the box for a 3 minute test phase with amount of time spent exploring each object recorded. The Discrimination ratio was calculated as an index of memory performance (time at novel−time at familiar)/(time at novel + time at familiar). A value of zero indicates no preference, whereas a positive value indicates preferential exploration of the novel configuration and a negative value indicates preferential exploration of the familiar configuration. For each mouse, the mean discrimination ratio for each task was obtained by calculating the mean of the discrimination ratios for the 2 trials on that task.

*11β-HSD activity assay*

11β-HSD1 and 2 activity was assessed in homogenates from E12.5 and E17.5 placentas and fetal heads. 11β-HSD2 activity was also measured in E12.5 and E17.5 , fetal bodies and adult kidneys. 11β-HSD2 assays were carried out as detailed in ([Kotelevtsev et al., 1999](#_ENREF_5)) using standardized amounts of protein for each tissue (fetal heads and bodies 4mg/ml; placenta and adult kidney 0.2mg/ml) incubated at 37°C with 400 μM NAD and 12 nM [^3^H]corticosterone. Fetal head and bodies were incubated for 2h and placenta and adult kidney incubated for 20 min. 11β-HSD1 activity assays were conducted as detailed in ([Nixon et al., 2012](#_ENREF_6)), homogenates (fetal heads 4mg/ml; placentas 0.2mg/ml) were incubated at 37°C with 2 mM NADP and 12 nM [^3^H]corticosterone. Fetal heads were incubated for 22 h and placenta for 1 h. Steroids were extracted after incubation with ethyl acetate, the organic phase was evaporated under nitrogen, and extracts were solubilized in the mobile phase (water to acetonitrile to methanol; 60:15:25, 1.5 mL/min). Steroids were separated by high-performance liquid chromatography using a C_18_ Sunfire column (Waters, Hertfordshire, U.K.) at 42°C and quantified by on-line liquid scintillation counting.

*CNS expression analyses*

In situ hybridisation was performed as described previously ([Harris et al., 2001](#_ENREF_4)). All in situ experiments were performed on fresh frozen brains and cryostat cut sections (10 μm) were collected. Briefly, plasmids containing fragments of cDNA for mouse serotonin receptor 1A (*Htr1a*), serotonin receptor 2C (*Htr2c*), tryptophan hydroxylase 2 *(Tph2)*, serotonin transporter (*Slc6a4*), dopamine receptor D2 (*Drd2*), dopamine transporter (*Slc6a3*), mineralocorticoid receptor (*Nr3c2*), glucocorticoid receptor (*Nr3c1*) and corticotrophin-releasing hormone (*Crh*) were used as templates to transcribe ^35^S-UTP radiolabelled antisense riboprobes. After hybridisation and stringent washes, the sections were exposed to autoradiographic film for 10 days (XAR-5; Kodak, Kemel Hempstead, UK). Slides were dipped in Kodak NTB2 emulsion, exposed at 4°C for 3 weeks, developed and counterstained (1% pyronin). Expression was quantified by counting silver grains overlying identified neurons under bright-field illumination using MCID basic software 7.0 (Interfocus Imaging Ltd., Linto, UK). No specific cellular hybridization signal was seen with any sense probe.

Expression of *Hsd11b2* and *Hsd11b1* in whole fetal brain and *Nr3c2* and *Nr3c1* in dissected hippocampi were assessed by quantitative real-time RT-PCR ([Wyrwoll et al., 2009](#_ENREF_8)) using Lightcycler 480 Probes Master (Roche Diagnostics, Burgess Hill, UK). The following primer sets (Invitrogen, Paisley, UK) were used; Hsd11b2: 5-CTGGGGTATCAAGGTCAGCATT-3 (forward) and 5-CGTGCTCAATGTAGTCTTCACCA-3 (reverse), Hsd11b1: 5- GAACTGCGTGACCTCTGTTCT-3 (forward) and 5- ACGTCAGCCACTACGATATTGA-3 (reverse), *Nr3c2*: 5-CCCTACCATGTCCTAGAAAAGC-3 (forward) and 5-AGAACGCTCCAAGGTCTGAG-3 (reverse), *Nr3c1*: 5-CAAAGATTGCAGGTATCCTATGAA-3 (forward) and 5-CTTGGCTCTTCAGACCTTCC-3 (reverse) and HPRT: 5-TCCTCCTCAGACCGCTTTT-3 (forward) CCTGGTTCATCATCGCTAATC (reverse).

*High performance liquid chromatography (HPLC)*

Brains were dissected into cortex, diencephalon, hippocampus and hindbrain the dissected samples were weighed and homogenised in 200µl 0.1M perchloric acid (PCA), sonicated twice for 10secs and spun (10000g, 5 mins, 4°C). The supernatant was extracted and 0.3µg/µl of internal standard (methyl-5-hydroxytryptamine, CH3-5HT, Sigma-Aldrich, Dorset, UK) was added before being filtered through a 22µm cellulose acetate centrifuge filter by application to a COSTARspinX column (Corning Life Sciences) and spun (10000g, 1 min, 4°C). A further 100µl 0.1M PCA was added to wash through the column and spun (10000g, 1 min, 4°C). Samples were kept on ice prior to chromatography and were run within 10 mins of preparation. Standards (serotonin creatinine sulphate (5-HT), 5-hydroxy-3-indole acetic acid (5HIAA), dopamine 3-hydroxytryptamine (DA), CH3-5HT, noradrenaline (NA) and homovanillic acid (HVA), all supplied by Sigma-Aldrich, Dorset, UK) and samples were loaded into a Waters 2695 separations module (Waters Corporation) which autoinjected 10µl into a Waters Sunfire 3.5µm C_18_ column (Waters Corporation) with mobile phase (6% methanol, 94% citrate buffer (pH3.5) filtered through 22µm nitrocellulose filter (Milllipore Corporation)) delivered at a flow rate of 0.4ml/min. Each sample took 35 mins to complete and the column maintained at 25°C. Natural fluorescence was detected by a Waters 2475 Multi λ Fluorescence Detector (Waters Corporation) at 284nm and 335nm. The area of the emission peak was used to determine the concentration of each molecule from the standard curve. This was then corrected for the recovery rate, determined by comparing the mean internal control areas obtained from the samples to the mean internal control areas obtained from standards. The concentration of each molecule in each sample was corrected by the tissue weight and volume of supernatant.

*Cardiovascular parameters*

Blood pressure and renal electrolyte excretion was measured in anaesthetized mice (Thiobutabarbital sodium (Inactin), 100 mg/kg; IP), as described ([Craigie et al., 2012](#_ENREF_3)). Briefly, mean arterial BP was measured continuously via a carotid cannula (Powerlab, AD Instruments, UK) over a 40-miunute period of urine collection. Urinary electrolytes were measured by ion selective electrode (Analyzer 9180, Roche, UK) and multiplied by urine flow to calculate excretion. At the end of the experiment, a 250μl sample of arterial blood was taken for measurement of haematocrit and plasma electrolyte concentrations.

**References**

Brown, R.W., Chapman, K.E., Edwards, C.R., Seckl, J.R., 1993. Human placental 11 beta-hydroxysteroid dehydrogenase: evidence for and partial purification of a distinct NAD-dependent isoform. Endocrinology 132, 2614-2621.

Cost, K.T., Lobell, T.D., Williams-Yee, Z.N., Henderson, S., Dohanich, G., 2014. The effects of pregnancy, lactation, and primiparity on object-in-place memory of female rats. Horm Behav 65, 32-39.

Craigie, E., Evans, L.C., Mullins, J.J., Bailey, M.A., 2012. Failure to downregulate the epithelial sodium channel causes salt sensitivity in Hsd11b2 heterozygote mice. Hypertension 60, 684-690.

Harris, H.J., Kotelevtsev, Y., Mullins, J.J., Seckl, J.R., Holmes, M.C., 2001. Intracellular regeneration of glucocorticoids by 11beta-hydroxysteroid dehydrogenase (11beta-HSD)-1 plays a key role in regulation of the hypothalamic-pituitary-adrenal axis: analysis of 11beta-HSD-1-deficient mice. Endocrinology 142, 114-120.

Kotelevtsev, Y., Brown, R.W., Fleming, S., Kenyon, C., Edwards, C.R., Seckl, J.R., Mullins, J.J., 1999. Hypertension in mice lacking 11beta-hydroxysteroid dehydrogenase type 2. J Clin Invest 103, 683-689.

Nixon, M., Wake, D.J., Livingstone, D.E., Stimson, R.H., Esteves, C.L., Seckl, J.R., Chapman, K.E., Andrew, R., Walker, B.R., 2012. Salicylate downregulates 11beta-HSD1 expression in adipose tissue in obese mice and in humans, mediating insulin sensitization. Diabetes 61, 790-796.

Tronche, F., Kellendonk, C., Kretz, O., Gass, P., Anlag, K., Orban, P.C., Bock, R., Klein, R., Schütz, G., 1999. Disruption of the glucocorticoid receptor gene in the nervous system results in reduced anxiety. Nature Genetics 23, 99-103.

Wyrwoll, C.S., Seckl, J.R., Holmes, M.C., 2009. Altered placental function of 11beta-hydroxysteroid dehydrogenase 2 knockout mice. Endocrinology 150, 1287-1293.

Yau, J.L., Noble, J., Kenyon, C.J., Hibberd, C., Kotelevtsev, Y., Mullins, J.J., Seckl, J.R., 2001. Lack of tissue glucocorticoid reactivation in 11beta -hydroxysteroid dehydrogenase type 1 knockout mice ameliorates age-related learning impairments. Proc Natl Acad Sci U S A 98, 4716-4721.

**Supplementary Results:**

**Tables**

**Table S1:** **Fetal growth parameters**

|  | CON | HSD2BKO |
| --- | --- | --- |
|  |  |  |
| Fetal weight (g):  E12.5  E17.5  Placental weight (g):  E12.5  E17.5 | 0.0746±0.0047 (7)  0.7930±0.0684 (5)  0.0589±0.0063 (7)  0.00916±0.0044 (6) | 0.0768±0.0026 (6)  0.8106±0.0294 (5)  0.0564±0.0017 (6)  0.0887±0.0023 (6) |
| Birth weight (g): | 1.429±0.032 (12) | 1.381±0.028 (15) |

No differences in fetal weight, placental weight or birthweight were apparent in HSD2BKO offspring in comparison to control littermates (n = 6-8)..

**Table S2:** **Neurodevelopment markers and cerebellar morphology.**

|  | CON | HSD2BKO |
| --- | --- | --- |
|  |  |  |
| Negative geotaxis  (% of offspring that turn to face upwards at P7) | 89 | 91 |
| Timing of eye opening  (% offspring with both eyes open at P14) | 68 | 63 |
| Cerebellar morphology (AU) |  |  |
| -total area | 57.2±3.46 | 52.8±5.93 |
| -molecular layer area | 28.8±4.21 | 26.3±2.64 |
| -granule layer area | 23.4±3.64 | 22.6±2.43 |

Neither differences in neurodevelopmental markers (n = 15), nor cerebellar morphology (n = 8) were apparent in HSD2BKO offspring in comparison to control littermates (n = 12 and n = 8, respectively).

**Table S3: High performance liquid chromatography (HPLC) of neurotransmitters in the diencephalon region of CON (n = 10) and HSD2BKO mice (n = 18).**

|  | CON | HSD2BKO |
| --- | --- | --- |
| **HPLC – diencephalon region** |  |  |
| 5-HT:5-HIAA | 3.43±0.041 | 3.409±0.016 |
| DA:HVA | 0.0634±0.014 | 0.0598±0.021 |
| DA:NA | 1.942±0.233 | 1.794±0.342 |

No significant changes were apparent in the levels of serotonin (5-HT), dopamine (DA) or their metabolites (5-hydroxy-3-indole acetic acid (5HIAA), homovanillic acid (HVA) and noradrenaline (NA).

**Supplementary Figures**

**Figure S1 Growth trajectory of HSD2BKO mice**

Body weight was unaltered between CON, HSD2BKO and NestinCre offspring until after weaning. Growth trajectory of the HSD2BKO offspring then closely followed that of NestinCre animals (n = 10-16).

**Figure S2 Examples of *Htr1a* mRNA expression in the hippocampus**

**A B C**


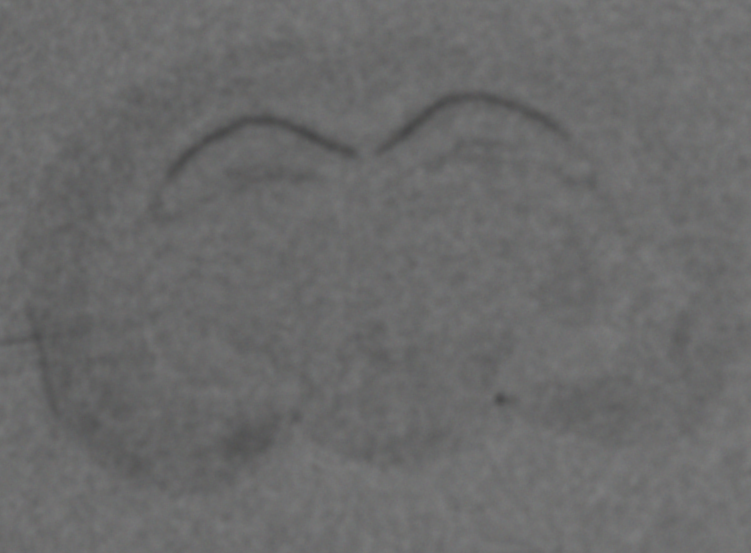

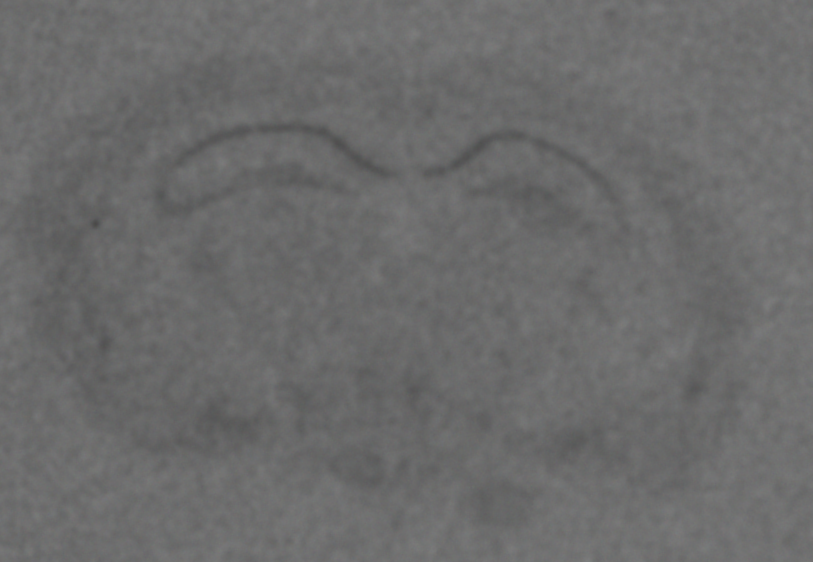

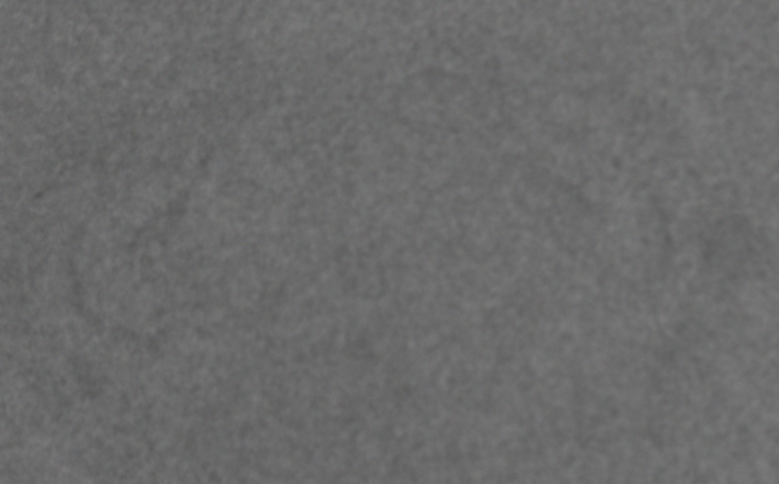


Control

(HSD2^flx/flx^)

HSD2BKO

(Nestin-cre.HSD2^flx/flx^)

Sense Control

(HSD2^flx/flx^)

In situ hybridisation of *Htr1a* mRNA expression in Control brain and brain from HSD2BKO mice using an antisense *Htr1a* riboprobe (A and B). A sense control is shown in C.
